# Resting-state functional connectivity is modulated by cognitive reserve in early Parkinson’s disease

**DOI:** 10.3389/fpsyg.2023.1207988

**Published:** 2023-08-25

**Authors:** Sonia Di Tella, Matteo De Marco, Francesca Baglio, Maria Caterina Silveri, Annalena Venneri

**Affiliations:** ^1^Department of Psychology, Università Cattolica del Sacro Cuore, Milan, Italy; ^2^IRCCS, Fondazione Don Carlo Gnocchi Onlus, Milan, Italy; ^3^Department of Life Sciences, Brunel University London, Uxbridge, United Kingdom; ^4^Department of Medicine and Surgery, University of Parma, Parma, Italy

**Keywords:** functional MRI, imaging, Parkinson’s disease, resting-state networks, cognitive reserve, brain reserve

## Abstract

**Background:**

Fronto-striatal disconnection is thought to be at the basis of dysexecutive symptoms in patients with Parkinson’s disease (PD). Multiple reserve-related processes may offer resilience against functional decline. Among these, cognitive reserve (CR) refers to the adaptability of cognitive processes.

**Objective:**

To test the hypothesis that functional connectivity of pathways associated with executive dysfunction in PD is modulated by CR.

**Methods:**

Twenty-six PD patients and 24 controls underwent resting-state functional magnetic resonance imaging. Functional connectivity was explored with independent component analysis and seed-based approaches. The following networks were selected from the outcome of the independent component analysis: default-mode (DMN), left and right fronto-parietal (l/rFPN), salience (SalN), sensorimotor (SMN), and occipital visual (OVN). Seed regions were selected in the substantia nigra and in the dorsolateral and ventromedial prefrontal cortex for the assessment of seed-based functional connectivity maps. Educational and occupational attainments were used as CR proxies.

**Results:**

Compared with their counterparts with high CR, PD individuals with low CR had reduced posterior DMN functional connectivity in the anterior cingulate and basal ganglia, and bilaterally reduced connectivity in fronto-parietal regions within the networks defined by the dorsolateral and ventrolateral prefrontal seeds. Hyper-connectivity was detected within medial prefrontal regions when comparing low-CR PD with low-CR controls.

**Conclusion:**

CR may exert a modulatory effect on functional connectivity in basal ganglia and executive-attentional fronto-parietal networks. In PD patients with low CR, attentional control networks seem to be downregulated, whereas higher recruitment of medial frontal regions suggests compensation via an upregulation mechanism. This upregulation might contribute to maintaining efficient cognitive functioning when posterior cortical function is progressively reduced.

## 1. Introduction

Among non-motor disturbances, cognitive impairment is common in individuals with Parkinson’s disease (PD). A growing body of evidence indicates that PD is hallmarked by cognitive decline in a range of cognitive domains. Decline of attentional and executive functioning is thought to result from disruption of striato-thalamo-frontal pathways, and is often a stable clinical trait already detectable at the earliest disease stages. Deficits of episodic memory and visuospatial skills, on the other hand, are related to dysfunction in temporo-parietal areas and tend to be more common when clinical decline leads to dementia (Williams-Gray et al., 2007; Papagno and Trojano, 2018).

Resting-state functional magnetic resonance imaging (rs-fMRI) holds considerable potential for the investigation of disruption of cognitive circuitries (Prvulovic et al., 2011; Ferreira and Busatto, 2013), by measuring temporal synchronisations in the blood-oxygen-level-dependent (BOLD) signal across brain regions at rest (Fox and Raichle, 2007). This has also been useful in the study of cognitive decline in PD (Wolters et al., 2019). This can be addressed by adopting complementary methodologies such as the extraction of large-scale functional networks (data-driven approach) or the calculation of functional connectivity (FC) maps based on an *a priori* seed region selection (theory-driven approach).

Several rs-fMRI studies have explored large-scale networks, reporting disruptions in the default-mode network (DMN) and in the fronto-parietal networks (FPNs) in PD with cognitive impairment (Lebedev et al., 2014; Madhyastha et al., 2015; Ruppert et al., 2021). Importantly, the brain regions that are part of these networks are areas involved in sensorimotor integration and in higher cognitive functioning. In healthy controls (HC), reduced DMN connectivity appears associated with decreased memory performance, slower processing speed and worse executive functioning (Andrews-Hanna et al., 2007; Damoiseaux et al., 2008; Vidal-Piñeiro et al., 2014). There has also been significant evidence of DMN disruption in other neurodegenerative disorders such as Alzheimer’s disease, Huntington’s disease and frontotemporal dementia (Zhou et al., 2010; Wolf et al., 2012; Balthazar et al., 2014). Similarly, changes in DMN connectivity have been previously reported in PD (Tessitore et al., 2012; Disbrow et al., 2014; Yao et al., 2014). Decreased FC within the DMN differentiates PD patients with and without cognitive impairment (Wolters et al., 2019). Tessitore et al. (2012) reported decreased DMN connectivity in the bilateral inferior parietal cortex in a cohort of cognitively unimpaired PD patients. They also showed significant positive correlations between DMN connectivity and cognitive performance in tests of memory and visuospatial functioning, suggesting that functional DMN alteration can precede objective cognitive impairment in PD (Tessitore et al., 2012).

The FPNs (also known as executive control networks) follow the dorsal-attentional streams (Fox et al., 2006) and support attentional control (Japee et al., 2015). These networks have intricate functional connections with the basal ganglia, in particular with the caudate nucleus (Seeley et al., 2007). Disruption in FPNs seems to have a critical role in determining cognitive decline in PD. Alterations in FPNs were reported in PD patients with cognitive impairment (Lewis et al., 2003; Caminiti et al., 2015). Furthermore, recent findings have demonstrated that the topological robustness of the FPNs is associated with the absence of cognitive decline in PD individuals, suggesting that the integrity of these networks may help support cognitive performance in PD (Cascone et al., 2021).

In the last decades, enormous progress has been made in understanding which factors may contribute to “resilience” against neurodegeneration. In this respect, reserve-related processes such as cognitive reserve (CR), brain reserve, and brain maintenance, which refers to the mitigation of age-related brain changes by life experiences, are known to play a major role in modulating neurofunctional resources (Stern, 2002; Stern et al., 2018). As initially observed in Alzheimer’s disease (Stern et al., 2018), these factors can account for the apparent lack of direct correspondence between the severity of pathological changes and the clinical manifestations; they might help the understanding of any differential susceptibility to the effects of pathology in PD, mainly in cognitive functions and functionality in daily-living activities.

CR refers to the processing resources accrued over time as a result of being engaged in mentally-stimulating activities, i.e., education, professional attainment, and leisure activities (Stern et al., 2018). To quantify CR, it is possible to rely on “convenience proxies” such as socio-behavioral indices, e.g., education, intelligence quotient, occupational complexity, leisure and physical activity (Stern et al., 2018).

Empirical evidence from studies of PD indicates that CR can modulate cognitive performance and contrast cognitive decline. Higher levels of education were found to be associated with better cognitive performance and slower cognitive decline (Hindle et al., 2014). A study of 35 non-demented PD patients, using the Cognitive Reserve Index questionnaire (Nucci et al., 2012) and the Brief Intelligence Test (Colombo et al., 2000), showed a meaningful and significant effect of CR on patients’ performance in tasks of executive function, the cognitive domain most affected in PD (Ciccarelli et al., 2018). Thus, a higher educational attainment, coupled with a high mentally-stimulating lifestyle, appears to support cognitive performance in PD and, therefore, limit cognitive deterioration.

To the best of our knowledge, to date no study has investigated the modulatory role of CR on FC in PD. Initial evidence from studies of HC suggests that education and CR might have a positive effect on FC networks. Arenaza-Urquijo et al. (2013) examined a cognitively healthy older cohort (60–80 years) and described better brain metabolism, larger gray matter (GM) volumes as well as enhanced FC in regions such as the anterior cingulate cortex, right hippocampus, right posterior cingulate cortex, left inferior frontal and left angular gyri in individuals with higher education (Arenaza-Urquijo et al., 2013). Similarly, Marques et al. (2015) examined the relationship between education and FC and found that individuals with higher education had wider connectivity networks in all lobes of both hemispheres. These authors suggested that increased connectivity might moderate the effects of age (Marques et al., 2015). Moreover, Marques et al. (2016), in a study of a cohort of 120 elderly HC, demonstrated that demographic characteristics (especially years of education) were associated with higher FC, in particular with higher clustering, local efficiency and strength in parietal and occipital regions. These findings, collectively, indicate that individuals with higher education rely on different neural processing (Marques et al., 2016). Amongst the main large-scale networks, it has been demonstrated that higher CR is associated with increased brain activity in the DMN in elderly HC (Bosch et al., 2010).

Further evidence of a modulatory role of CR on FC has been obtained from individuals with other neurodegenerative conditions (Bozzali et al., 2015; Franzmeier et al., 2017a,b; Fuchs et al., 2019). Bozzali et al. (2015) investigated whether CR modulates FC in healthy, amnestic mild cognitive impairment, and Alzheimer’s disease individuals. The authors found that individuals with Alzheimer’s disease and higher education levels had greater FC in the DMN compared with individuals with Alzheimer’s disease and lower education levels (Bozzali et al., 2015). Some of the amnestic mild cognitive impairment patients had similar connectivity strength, suggesting that education and, more in general, CR, fosters mechanisms of compensation and limits progression of atrophy. A pioneering study in patients with multiple sclerosis used premorbid verbal intelligence as a proxy for CR and network-based measures to demonstrate that patients with higher CR had more preserved FC despite having GM atrophy (Fuchs et al., 2019). These authors hypothesized that preservation of network FC attenuates the impact of structural network disruption on cognition (in particular on cognitive processing speed and visual/spatial memory) in patients with multiple sclerosis.

This study tested the hypothesis that, in PD patients, FC alterations can be detected in large-scale and seed-based resting-state brain networks. It also tested the hypothesis that the patterns of alteration would be modulated by CR. First, we explored if CR is associated with the activity of the main large-scale functional cognitive networks, namely the anterior and posterior DMN (aDMN, pDMN, respectively), the left and right FPN (lFPN, rFPN, respectively) and the salience network (SalN) in a group of PD patients and one of HC. The DMN and SalN were chosen based on their well-established association with cognitive performance (Esposito et al., 2009; Menon and Uddin, 2010). FPNs were selected because of their online role in executive control (Zanto and Gazzaley, 2013). We also tested the impact of CR on two additional large-scale networks: the sensorimotor network (SMN) and the occipital visual network (OVN). The SMN was selected because of its documented disruption in PD and because it is typically associated with cardinal motor symptoms (Tessitore et al., 2014). The OVN was instead chosen as a non-cognitive control network. Second, we explored if CR modulates FC of key seed regions (dorsolateral and ventrolateral prefrontal cortex and substantia nigra) in PD and HC groups. We hypothesized that FC would be reduced in PD patients more than in HC in fronto-parietal regions, and that alterations of FC would be greater in patients with low CR.

## 2. Materials and methods

### 2.1. Participants

Fifty right-handed participants were included: 26 PD patients and 24 age-matched HC. Sample size was determined based on widely accepted and validated sample size minimums for fMRI studies (Desmond and Glover, 2002; Szucs and Ioannidis, 2020). Patients inclusion criteria were: diagnosis of idiopathic PD according to the Movement Disorder Society Clinical Diagnostic Criteria for PD (Postuma et al., 2015); positive DaTscan; mild-to-moderate disease stage (Modified Hoehn and Yahr, range 1–2) (Goetz et al., 2004; Postuma et al., 2015); stable therapy with either L-Dopa or dopamine agonists; absence of on–off fluctuations and dyskinesias due to medication. Exclusion criteria were: clinical signs meeting criteria for other neurological disorders, including atypical and iatrogenic parkinsonism; major psychiatric disorders. Although this was not explicitly recorded, at the time of the study the majority of the participants was retired.

All PD patients underwent a neurological examination and a neuropsychological assessment (Table 1). HC completed a neurological screening to rule out neuropsychiatric disorders, systemic and neurological diseases.

**Table 1:** Demographic and neurostructural characteristics of the cohort.

	HC [*N* = 24]	PD [*N* = 26]	Group comparison [*p* value]
Age [years, mean (SD)]	64.81 [7.95]	65.24 [8.07]	0.851^b^
Education [years, mean (SD)]	15.25 [4.17]	13.46 [4.47]	0.151^b^
Gender (Males/Females, *n*)	17/7	17/9	0.680^a^
MoCA [mean (SD)]	26.20 [2.78]	23.99 [3.08]	**0.025^b^**
Cognitive Reserve Composite Index [median (IQR)]	8.00 [3.75]	7.00 [5.00]	0.255^c^
Cognitive Reserve Index Global Score [mean (SD)]	126.36 [19.87]	130.13 [23.24]	0.148^b^
Cognitive Reserve Index Education [mean (SD)]	114.24 [15.38]	121.04 [16.52]	0.173^b^
Cognitive Reserve Index Working [mean (SD)]	116.44 [22.51]	123.96 [14.54]	0.476^b^
Cognitive Reserve Index Leisure [mean (SD)]	129.08 [23.95]	123.17 [32.00]	0.551^b^
Gray matter volume [ml, mean (SD)]	635.39 [64.50]	615.43 [68.30]	0.294^b^
White matter volume [ml, mean (SD)]	467.14 [59.75]	471.42[70.53]	0.819^b^
Cerebro-spinal fluid [ml, mean (SD)]	423.08 [96.43]	437.50 [72.90]	0.552^b^
Total intracranial volume [ml, mean (SD)]	1525.62 [133.94]	1524.35 [156.19]	0.976^b^
H & Y [median (IQR)]		1.50 [1.00]	
MDS-UPDRS III [median (IQR)]		20.00 [16.00]	
LEDD [mean (SD)]		247.84 [186.99]	
Disease duration [years, mean (SD)]		3.12 [2.12]	
Phonological Fluency [mean (SD)]		35.83 [9.20]	
Semantic Fluency [mean (SD)]		42.81 [8.58]	
TMT part A [mean (SD)]		46.08 [24.15]	
TMT part B [mean (SD)]		99.19 [89.12]	
TMT part B-A [mean (SD)]		55.38 [72.81]	
Rey-Osterrieth Figure Copy (0–36) [mean (SD)]		30.73 [5.56]	
Rey-Osterrieth Figure Recall (0–36) [mean (SD)]		15.40 [6.26]	
FCSRT IFR (0–36) [mean (SD)]		29.03 [3.85]	
FCSRT ITR* (0–36) [mean (SD)]		35.71 [0.69]	
FCSRT DFR (0–12) [mean (SD)]		10.45 [1.29]	
FCSRT DTR* (0–12) [mean (SD)]		11.96 [0.20]	
FCSRT ISC (0–1) [mean (SD)]		0.91 [0.28]	
FCSRT number of intrusions [mean (SD)]		0.04 [0.20]	
Raven Colored Matrices (0–36) [mean (SD)]		29.63 [4.73]	

For PD patients, a detailed neuropsychological assessment is reported. All these scores were above (or below, for response times and error scores) the cut-offs reported by Italian normative studies. Adjusted or raw scores (*indicates where raw scores are reported) are included. ^a^Chi-squared (*χ*^2^) test, ^b^Independent-sample Student’s *t*-test and ^c^Mann–Whitney’ s *U* test were used to evaluate group differences, as appropriate. *p*-values lower than 0.05 were considered significant and are highlighted in bold. CSI, Cueing Sensitivity Index; DFR, Delayed Free Recall; DTR, Delayed Total Recall; FCSRT, Free and Cued Selective Reminding Test; H & Y, Modified Hoehn and Yahr Scale; IFR, Immediate Free Recall; ITR, Immediate Total Recall; LEDD, Levodopa Equivalent Daily Dose; MoCA, Montreal Cognitive Assessment; SD, standard deviation; TMT, Trail Making Test; UPDRS III, Modified version of the Unified Parkinson’s Disease Rating Scale–motor part III.

Montreal Cognitive Assessment (MoCA) was used to screen for global cognitive status of all recruited participants to exclude frank dementia. A cut-off score of 15.5 was used for this purpose, based on Italian normative data (Santangelo et al., 2015).

Information on educational and occupational attainment was collected from all participants to compute a composite CR index, following the procedure described by Garibotto et al. (2008). Each patient was assigned to one of the following six occupational categories, associated with an incremental score from 1 to 6: (1) no occupation; (2) unskilled laborer; (3) stay-at-home spouse/partner; (4) skilled laborer, tradesman, lower-level civil servant, employee, self-employed small business, office or sales personnel; (5) mid-level civil servant or management, head of a small business, academician or specialist in a subordinate position; (6) senior civil servant or management, senior academic position, self-employed with high degree of responsibility. To balance the weight of the proxies, a six-rank transformation was applied to the distribution of years of educational attainment across the whole sample. Summative CR composites were calculated adding up education and occupation-related scores. The median was calculated for this composite index to split the cohort into subgroups of high and low CR. Consequently, four subgroups were defined: low-CR and high-CR PD patients; low-CR and high-CR HC.

Participants were also asked to complete the Cognitive Reserve Index questionnaire - CRIq (Nucci et al., 2012) to obtain a more detailed CR profile.

All participants provided written informed consent. The study was approved by the IRCCS Don Carlo Gnocchi Foundation Ethics Committee (3_1/7/2015).

### 2.2. MRI acquisition

All participants underwent a brain MRI scan acquired with a 1.5 T Siemens Avanto scanner equipped with a 12-channel head coil. The acquisition protocol comprised: (1) dual-echo turbo-spin echo proton-density/T2-weighted sequence [repetition time (TR) = 5,550 ms, echo time (TE) = 23/103 ms, matrix size = 320 × 320 × 45, resolution 0.8 × 0.8 × 3 mm^3^] to exclude patients showing any macroscopic brain lesions or white-matter hyperintensities, i.e., one or more macroscopic deep-white matter hyperintensities and/or more than five periventricular hyperintensities (Vale et al., 2015); (2) 3D high-resolution magnetisation-prepared rapid gradient echo (MPRAGE) T1-weighted image [TR = 1,900 ms, TE = 3.3 ms, inversion time (TI) = 1,100 ms, matrix size = 192 × 256 × 176, resolution = 1 mm^3^ isotropic]; (3) rs-fMRI sequence (TR = 2,570 ms, TE = 34 ms, matrix size = 64 × 64 × 31, resolution = 3.75 × 3.75 × 4.5 mm^3^). One 200-volume run of contiguous axial slices acquired in interleaved order was obtained for each participant. Prior to MRI acquisition, all participants were instructed to lay supine and keep their eyes closed without falling asleep for the full duration of the scan.

### 2.3. MRI data pre-processing

Pre-processing of functional data was completed with Statistical Parametric Mapping (SPM) 12 (Wellcome Centre for Human Neuroimaging, London, United Kingdom) implemented in MATLAB R2014a (Mathworks Inc., United Kingdom).

Scans were initially slice-timed (Sladky et al., 2011) and realigned (Friston et al., 1996) to correct for intra-volume temporal displacement and inter-volume spatial dislocation. Plots of linear and rotational in-scanner motion were visually inspected to rule out the presence of major artifacts. A 3-mm (or 3-degree) threshold was chosen as limit of acceptable motion (Damoiseaux et al., 2008; De Flores et al., 2017; Icenhour et al., 2017; Lazarov et al., 2017; Onoda et al., 2017). Realigned images were then spatially normalized and registered to the Montreal Neurological Institute (MNI) space, and voxel size was isotropied to 2 mm^3^.

Next, in order to remove part of non-neuronal contributions to the BOLD signal mostly due to physiological fluctuations, i.e., respiration and cardiac pulsation (Cordes et al., 2001), the REST toolbox^1^ was used to band-pass filter at 0.01–0.1 Hz the normalized images that were subsequently smoothed with a 6-mm full-width at half maximum Gaussian kernel (Friston et al., 2000).

### 2.4. Voxel-based morphometry analysis

T1-weighted structural images were also pre-processed to analyze global neurovolumetric properties, as per the most updated version of standard Voxel-Based Morphometry (VBM) methodology (Ashburner and Friston, 2000). This procedure includes probabilistic tissue-class segmentation (GM, white matter, and cerebrospinal fluid) in the MNI space, and a spatial smoothing with an 8 mm^3^ full-width at half maximum Gaussian kernel. Finally, a quantification of tissue-class maps in the subject-specific native space was carried out using the “get_totals” command line^2^, and total intracranial volumes were computed by summing up the volume of all tissue classes.

### 2.5. fMRI processing: independent component analysis networks

The first approach to the analyses processes the spatial outline of a set of maps generated with an independent component analysis (ICA), a technique that analyzes the whole fMRI dataset, separating signal and noise into a selected number of latent variables (components), each of which embodies an independent source of signal and has its own topography (Fox and Raichle, 2007).

The ICA fMRI toolbox GIFT (v1.3i)^3^ was used in combination with the Infomax optimization principle, and the number of components to be extracted was set at 20, as proficiently done by landmark research (Biswal et al., 2010; Kalcher et al., 2012).

Networks of interest were then identified based on their spatial outline (Roquet et al., 2014) and, of these, five were selected given their involvements in cognitive performance and executive control. These were the aDMN and pDMN (Esposito et al., 2009), the lFPN and rFPN (Zanto and Gazzaley, 2013), and the SalN (Menon and Uddin, 2010). The SMN, disrupted in PD (Tessitore et al., 2014), and one further non-cognitive control network, the OVN, were also considered.

### 2.6. fMRI processing: seed-based FC networks

**Figure 1:**
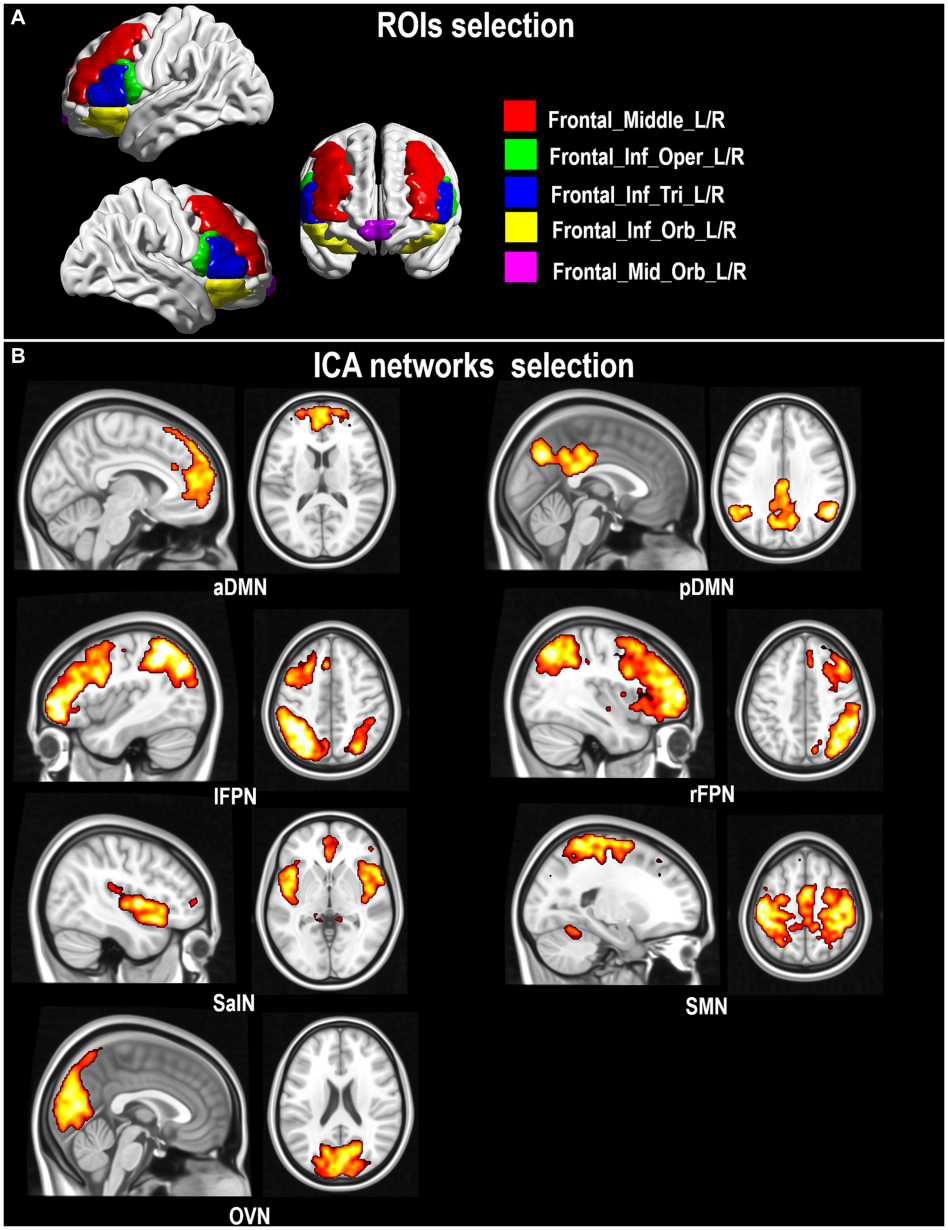
Seeds of interest in the dorsolateral and ventrolateral prefrontal cortex **(A)** and extracted rs-fMRI networks **(B)**.

A seed-based approach was also implemented with an *a priori* choice of seeds of interest relevant to PD pathophysiology. The focus was on those regions of the frontal lobe that receive dopaminergic innervations from the striatum, and that might thus benefit from CR in individuals with PD (Owen, 2004). Binary seed masks were created using the PickAtlas toolbox (Maldjian et al., 2003) based on anatomical landmarks identified within the IBASPM-116 Atlas^4^ and the CIT168 Reinforcement Learning Atlas^5^ (Pauli et al., 2018). The following regions were defined, maintaining left and right seeds separated: dorsolateral prefrontal cortex (middle frontal gyrus, “*Frontal_Mid_L/R*”), ventrolateral prefrontal cortex (inferior frontal gyrus, “*Frontal_Inf_Oper_L/R*”, “*Frontal_Inf_Tri_L/R*”; “*Frontal_Inf_Orb_L/R*”), ventromedial prefrontal cortex (VMPFC, “*Frontal_Mid_Orb_L/R*”), substantia nigra (combining pars compacta “*SNc*” and pars reticulata “*SNr*”: “*SNc + r*”). Selected frontal ROIs are illustrated in Figure 1A.

Seed-based timecourses were extracted from each region using the MarsBAR toolbox (Brett et al., 2002). FC maps were obtained for each participant by modeling the linear association between seed timecourse and the timecourse of each voxel of the brain. Timecourses extracted from the maps of white matter and cerebrospinal fluid were inserted in the model as nuisance regressors, in addition to the six translational and rotational rigid-body motion parameters, their squared values, their temporal derivatives, and the square derivatives.

### 2.7. Statistical analysis

Group differences in demographic and clinical variables were assessed with ANOVAs, chi-squared (*χ^2^*) tests, independent samples *t*-tests, or Mann–Whitney *U* tests, as appropriate. One-sample *t*-test models were initially run on all 50 normalized maps to identify the regional contour of the seven ICA-derived networks.

Group-level inferential models were run to compare the seven targeted functional networks (aDMN, pDMN, lFPN, rFPN, SalN, SMN, OVN) and the ten (four left, four right and two bilateral) maps of seed-based connectivity between HC and PD participants with low and high CR. A 2 × 2 ANCOVA full-factorial model was run to investigate the main effect of ‘group’ (HC, PD) and ‘CR’ (high, low) and their interaction on each functional map. Age was used as covariate. Following significant interactions, *post-hoc* comparisons were run to describe group differences in detail.

Modeling of GM maps served to test for the presence of regional neurostructural differences between diagnoses. An ANCOVA model comparable to that described above was also tested on GM. Total intracranial volume was included as a second covariate in this latter analysis to control for a global index of brain reserve.

Cluster-forming threshold of significance was set at *p* < 0.005 (uncorrected). Only clusters surviving a cluster-level *p_FWE_* < 0.05 were reported as significant.

MNI coordinates were converted into Talairach space via a non-linear transformation^6^, and were interpreted with the Talairach Daemon Client (Lancaster et al., 2000).

## 3. Results

### 3.1. Demographic and neurostructural measures

HC and PD groups did not differ in age, years of education, gender or global volumetric brain measurements (Table 1, see Supplementary Table S1 in Supplementary material for a detailed view on each diagnostic group split by CR). A significant difference was observed in global level of cognitive functioning (MoCA test), although no participant performed under the cut-off. No group difference was observed when CR was compared. The median CR was 7 in the whole sample (range: 3–11). Based on the median, four subgroups were identified as follows: 15 low-CR and 11 high-CR PD patients; 10 low-CR and 14 high-CR HC. No differences emerged in any of the demographic or neurostructural measures across the four subgroups. Although no difference in any of the clinical measures was observed between the two PD subgroups, patients with higher CR showed slightly less motor impairment, as evaluated with the Movement Disorder Society Unified Parkinson’s Disease Rating Scale (MDS-UPDRS) III (see Supplementary Table S2 in Supplementary material). CRIq data were available for 48 participants (Table 1). All CRIq subscores were significantly correlated with the CR composite scores (all *rho* coefficients >0.47).

### 3.2. Inferential models on ICA networks

One-sample *t*-tests (thresholded at a cluster-level *p_FWE_* < 0.05) were run across the whole cohort to visualize the target functional networks (Figure 1B).

A significant effect of ‘group’ was found in several resting-state functional networks (Table 2). PD patients showed less FC within the aDMN in the right primary motor (BA4), somatosensory and superior parietal cortices (BA3 and BA5), and within the SMN in the right premotor and supplementary motor areas (BA6). PD patients also showed significantly more FC within the rFPN in the right inferior frontal gyrus (BA47), anterior cingulate (BA32), and caudate nucleus.

**Table 2:** ANCOVA ‘group’ (HC, PD) and ‘CR’ (high, low) results of the ICA-extracted rs-fMRI networks: main effects of group and *post-hoc* tests for the interaction effects.

	Cluster	Peak	Peak	[mm]					
	Extent	*T*	equivZ	*x*	*y*	*z*	Side	Region	BA
Main effect of group: HC > PD									
aDMN									
	235	4.54	4.10	16	−40	66	R	Postcentral Gyrus	BA 3
		4.30	3.91	6	−40	60	R	Paracentral Lobule	BA 4
		4.04	3.71	10	−42	66	R	Paracentral Lobule	BA 4
		3.84	3.55	18	−20	66	R	Precentral Gyrus	BA 6
		3.65	3.40	18	−34	66	R	Postcentral Gyrus	BA 4
		3.64	3.39	22	−44	62	R	Superior Parietal Lobule	BA 5
		3.36	3.16	12	−48	62	R	Paracentral Lobule	BA 5
		3.19	3.01	16	−28	62	R	Precentral Gyrus	BA 4
SMN									
	170	4.08	3.75	8	0	64	R	Medial Frontal Gyrus	BA 6
		3.93	3.62	0	−14	70	R	Medial Frontal Gyrus	BA 6
		3.87	3.58	14	−8	64	R	Medial Frontal Gyrus	BA 6
		3.77	3.50	8	−20	66	R	Superior Frontal Gyrus	BA 6
		3.65	3.40	2	−20	60	R	Medial Frontal Gyrus	BA 6
Main effect of group: PD > HC									
RFPN									
	399	4.80	4.29	38	24	2	R	Inferior Frontal Gyrus	BA 47
		4.09	3.75	50	28	−4	R	Inferior Frontal Gyrus	BA 47
		4.01	3.69	54	30	−2	R	Inferior Frontal Gyrus	BA 47
		3.73	3.47	16	38	2	R	Anterior Cingulate	BA 32
		3.56	3.32	32	30	−4	R	Inferior Frontal Gyrus	BA 47
		3.53	3.29	16	26	−2	R	Caudate Nucleus	
		3.28	3.09	28	30	2	R	Inferior Frontal Gyrus	BA 47
*Post-Hoc* interaction Low CR: HC > PD									
pDMN	176	5.22	4.16	6	22	14	R	Anterior Cingulate	BA 24
		5.15	4.13	−4	22	14	L	Anterior Cingulate	BA 24
		4.82	3.94	22	20	8	R	Putamen	
		3.26	2.91	6	12	8	R	Caudate Nucleus	

*P*-values (*FWE* corrected) lower than 0.05 were considered significant. BA, Brodmann area; *x*, *y*, *z*, coordinates in the Montreal Neurological Institute (MNI) space.

**Figure 2:**
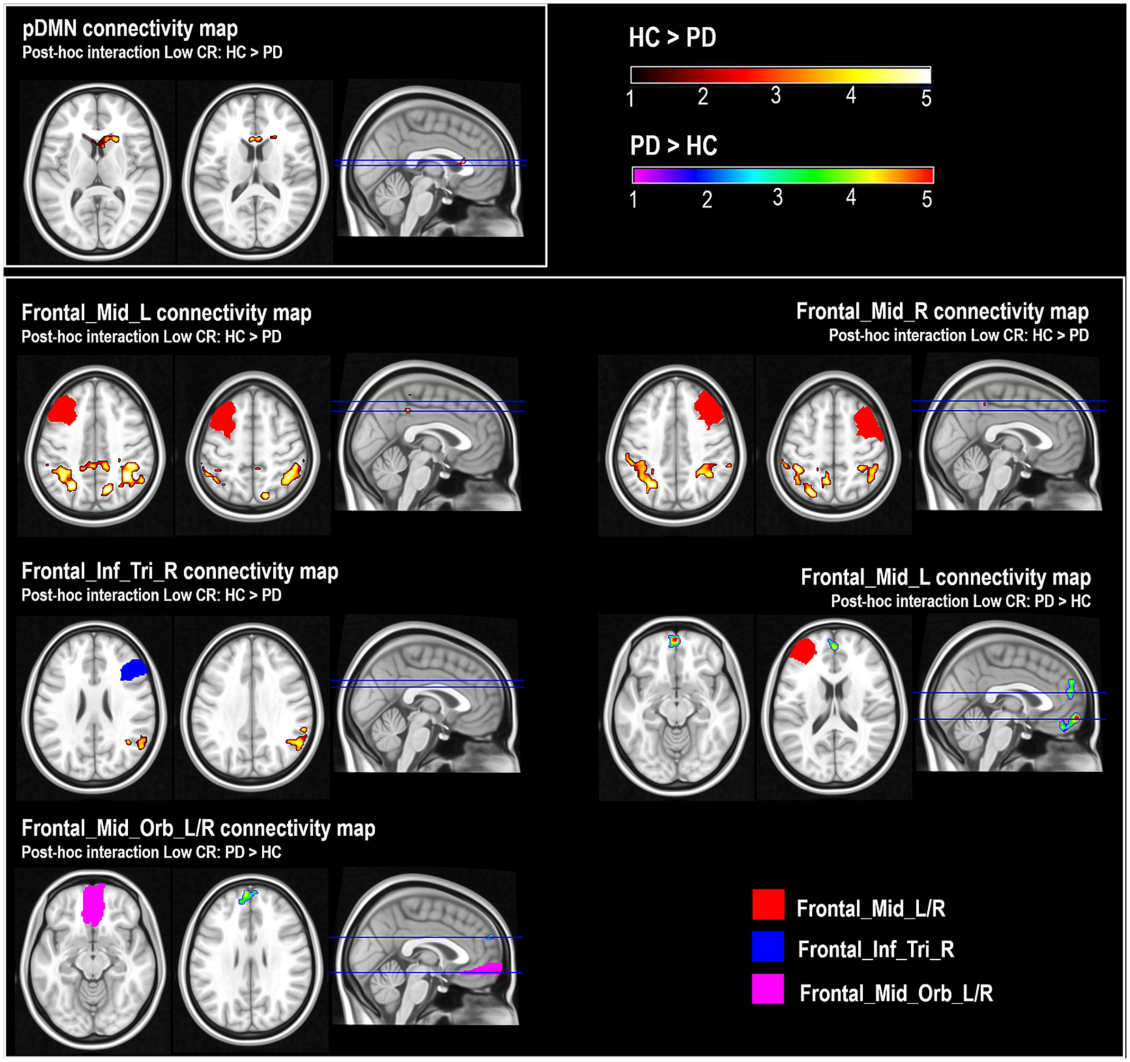
*Post-hoc* comparisons of pDMN (posterior Default Mode Network) maps and connectivity maps of ‘*Frontal_Mid_L’*, ‘*Frontal_Mid_R*’ and ‘*Frontal_Inf_Tri_R*’ between healthy controls (HC) with low CR and patients with Parkinson’s disease (PD) with low CR (Low-CR: HC > PD); and *post-hoc* comparisons of connectivity maps of ‘*Frontal_Mid_L*’ and ‘*Frontal_Mid_Orb_L_R*’ between HC with low CR and PD patients with low CR (Low-CR: PD > HC). Color scales represent the z-score associated with the statistical model. Although some of the clusters (e.g., the cluster emerging as part of the analysis of the pDMN) include a portion of voxels located in white matter, the core of the findings was located in the gray-matter submap.

A significant ‘group-by-CR’ interaction was found in the connectivity of the pDMN. This effect indicated that the “low-CR disadvantage” (represented by the ‘high-CR > low-CR’ contrast) was significantly stronger in individuals with PD. This was confirmed by a *post hoc* model analyzing the ‘low-CR HC > low-CR PD’ contrast. The effect was found in a small region extending from the basal ganglia (putamen and caudate nucleus) to the anterior cingulate (BA24, Table 2; Figure 2).

### 3.3. Inferential seed-based models

A significant effect of ‘group’ was found in several maps of seed-based FC (Table 3). PD patients had less FC than HC between the “*Frontal_Mid_L/R*” seed and parietal areas bilaterally (BA7 and BA40), precuneus and posterior cingulate (BA31), and between “*Frontal_Inf_Oper_R*” and the left insula (BA13) and inferior parietal lobule (BA40). In the PD group less FC was also detected between “*Frontal_Mid_Orb_L*” and the right fusiform (BA19) and inferior occipital gyri (BA19 and BA18).

**Table 3:** ANCOVA ‘group’ (HC, PD) and ‘CR’ (high, low) results of the maps of seeds of interest: main effects of group and *post-hoc* tests for the interaction effects.

	Cluster	Peak	Peak	[mm]					
	Extent	*T*	equivZ	*x*	*y*	*z*	Side	Region	BA
Main effect of group: HC > PD									
Frontal_Mid_L									
	889	6.27	5.29	36	−52	44	R	Inferior Parietal Lobule	BA 40
		4.85	4.33	54	−40	42	R	Inferior Parietal Lobule	BA 40
		4.61	4.15	64	−40	36	R	Inferior Parietal Lobule	BA 40
	434	4.79	4.28	−28	−62	42	L	Superior Parietal Lobule	BA 7
		4.48	4.05	−36	−48	38	L	Inferior Parietal Lobule	BA 40
		4.05	3.72	−20	−56	34	L	Posterior Cingulate Gyrus	BA 31
Frontal_Mid_R									
	790	5.84	5.01	−40	−42	54	L	Inferior Parietal Lobule	BA 40
		4.51	4.07	−28	−62	40	L	Superior Parietal Lobule	BA 7
		4.35	3.96	−40	−34	42	L	Inferior Parietal Lobule	BA 40
	327	5.56	4.82	38	−50	38	R	Inferior Parietal Lobule	BA 40
		4.18	3.82	40	−40	46	R	Inferior Parietal Lobule	BA 40
		3.65	3.40	44	−46	52	R	Inferior Parietal Lobule	BA 40
	333	4.27	3.89	−20	−32	60	L	Postcentral Gyrus	BA 3
		4.14	3.79	−20	−46	66	L	Superior Parietal Lobule	BA 5
		3.80	3.52	−10	−62	48	L	Precuneus	BA 7
Frontal_Inf_Oper_R									
	732	5.04	4.46	−32	−42	22	L	Insula	BA 13
		4.48	4.06	−46	−36	34	L	Inferior Parietal Lobule	BA 40
		4.34	3.95	−50	−44	50	L	Inferior Parietal Lobule	BA 40
Frontal_Mid_Orb_L									
	261	5.05	4.47	40	−72	−16	R	Fusiform Gyrus	BA 19
		3.34	3.14	36	−78	−12	R	Inferior Occipital Gyrus	BA 19
		3.29	3.09	32	−86	−12	R	Inferior Occipital Gyrus	BA 18
Main effect of group: PD > HC									
Frontal_Mid_L									
	242	5.41	4.72	−18	−52	−4	L	Lingual Gyrus	BA 19
		4.88	4.35	−18	−60	2	L	Posterior Cingulate Gyrus	BA 30
		3.42	3.21	−12	−44	4	L	Posterior Cingulate Gyrus	BA 29
Frontal_Mid_R									
	416	5.07	4.49	−22	−90	20	L	Cuneus	BA 18
		4.03	3.70	−14	−96	18	L	Cuneus	BA 18
		3.87	3.57	−38	−80	12	L	Middle Temporal Gyrus	BA 39
Frontal_Inf_Tri_R									
	304	5.60	4.85	−18	−80	−16	L	Lingual Gyrus	BA 18
		4.31	3.92	−14	−92	0	L	Cuneus	BA 17
		3.67	3.42	−6	−90	8	L	Cuneus	BA 18
	466	4.79	4.28	24	−84	−6	R	Middle Occipital Gyrus	BA 18
		4.51	4.08	30	−86	16	R	Middle Occipital Gyrus	BA 19
		4.16	3.80	20	−96	6	R	Cuneus	BA 18
Main effect of CR: Low CR > High CR									
L_Mid_Front									
	275	4.65	4.18	46	−60	−28	R	Cerebellum Declive	
		3.78	3.50	48	−60	−38	R	Cerebellum Tuber	
		3.55	3.32	28	−68	−44	R	Cerebellum Pyramis	
		3.49	3.27	38	−62	−38	R	Cerebellum Tuber	
		3.21	3.03	42	−58	−48	R	Cerebellum Cerebellar Tonsil	
		4.65	4.18	46	−60	−28	R	Cerebellum Declive	
*Post-Hoc* interaction Low CR: HC > PD									
Frontal_Mid_L									
	1,302	7.81	5.35	44	−46	48	R	Inferior Parietal Lobule	BA 40
		6.67	4.88	36	−50	40	R	Inferior Parietal Lobule	BA 40
		5.48	4.3	40	−38	44	R	Inferior Parietal Lobule	BA 40
	998	7.31	5.15	−34	−46	38	L	Inferior Parietal Lobule	BA 40
		6.11	4.62	−26	−60	40	L	Superior Parietal Lobule	BA 7
		5.27	4.19	−52	−38	36	L	Inferior Parietal Lobule	BA 40
	297	5.79	4.46	16	−66	40	R	Precuneus	BA 7
		5.36	4.24	12	−70	46	R	Precuneus	BA 7
		3.64	3.18	14	−58	48	R	Precuneus	BA 7
	299	5.51	4.32	14	−42	38	R	Cingulate Gyrus	BA 31
		5.38	4.26	18	−52	28	R	Precuneus	BA 31
		4.06	3.47	−10	−40	44	L	Cingulate Gyrus	BA 31
Frontal_Mid_R									
	377	6.23	4.68	−8	−56	54	L	Precuneus	BA 7
		5.98	4.56	−20	−46	68	L	Postcentral Gyrus	BA 5
		4.27	3.60	−22	−30	68	L	Postcentral Gyrus	BA 3
	1,561	6.03	4.58	−50	−38	36	L	Inferior Parietal Lobule	BA 40
		5.97	4.56	−36	−44	30	L	Supramarginal Gyrus	BA 40
		5.73	4.43	−42	−40	54	L	Inferior Parietal Lobule	BA 40
	657	5.32	4.22	38	−50	38	R	Inferior Parietal Lobule	BA 40
		4.60	3.81	32	−42	52	R	Inferior Parietal Lobule	BA 40
		4.44	3.71	30	−46	42	R	Precuneus	BA 7
Frontal_Inf_Tri_R									
	544	5.42	4.28	58	−42	36	R	Supramarginal Gyrus	BA 40
		5.19	4.15	54	−58	36	R	Inferior Parietal Lobule	BA 40
		4.28	3.61	52	−56	22	R	Superior Temporal Gyrus	BA 39
*Post-Hoc* interaction Low CR: PD > HC									
Frontal_Mid_L									
	366	5.77	4.45	0	62	−12	L	Medial Frontal Gyrus	BA 11
		4.66	3.84	2	46	−22	R	Orbital Gyrus	BA 11
		4.12	3.51	−8	38	−16	L	Medial Frontal Gyrus	BA 11
	200	4.71	3.88	−22	34	36	L	Superior Frontal Gyrus	BA 9
		4.45	3.72	−8	44	40	L	Medial Frontal Gyrus	BA 8
		3.91	3.37	−14	32	36	L	Medial Frontal Gyrus	BA 9
	196	4.48	3.73	0	52	18	L	Medial Frontal Gyrus	BA 9
		4.01	3.44	0	54	30	L	Superior Frontal Gyrus	BA 9
		3.12	2.81	−6	60	20	L	Medial Frontal Gyrus	BA 10
Frontal_Mid_Orb_L/R									
	208	4.71	3.88	−6	60	24	L	Medial Frontal Gyrus	BA 10
		4.00	3.43	−16	56	24	L	Superior Frontal Gyrus	BA 10
		3.92	3.38	−14	44	36	L	Superior Frontal Gyrus	BA 9

*P*-values (*FWE* corrected) lower than 0.05 were considered significant. Legend: BA – Brodmann area; *x*, *y*, *z* – coordinates in the Montreal Neurological Institute (MNI) space.

In contrast, PD patients had more FC between “*Frontal_Mid_L*” and the left lingual gyrus (BA19) and posterior cingulate (BA30 and BA29), between “*Frontal_Mid_R*” and the left cuneus (BA19) and middle temporal gyrus (BA39), and between “*Frontal_Inf_Tri_R*” and bilateral posterior areas covering the lingual, middle occipital and cuneal regions (BA17, BA18 and BA19). Patients and HC showed no significant differences in the FC pattern of “*SNc + r*”.

A significant effect of ‘CR’ was found in the map of “*Frontal_Mid_L*”, with low-CR individuals showing more FC between “*Frontal_Mid_L*” and the right cerebellum.

A significant ‘group-by-CR’ interaction, indicating a “low-CR disadvantage” statistically stronger in the group of PD individuals, was also found. This, again, emerged when low-CR participants were analyzed at *post hoc*, with low-CR HC showing more FC than low-CR PD between the “*Frontal_Mid_L/R*” and the bilateral inferior parietal lobule (BA40), precuneus (BA7) and posterior cingulate (BA31), and between “*Frontal_Inf_Tri_R*” and a region including part of the right supramarginal, superior temporal gyri and inferior parietal lobule (BA39 and BA40). No differences emerged in the subgroups of participants with high-CR (Table 3; Figure 2).

The opposite interaction contrast (indicating a weaker “low-CR disadvantage” in participants with PD) also yielded significant results. *Post-hoc* comparisons revealed that low-CR PD had higher FC than low-CR HC between “*Frontal_Mid_L*” and the medial and superior frontal gyri (BA8, BA9, BA10 and BA11), and between “*Frontal_Mid_Orb_L_R*” and the medial and superior frontal gyri (BA10 and BA 9). No differences were found in the subgroups of participants with high CR (Table 3; Figure 2).

To evaluate differences in in-scanner motion between the two diagnostic groups, individual framewise displacement values were computed (Power et al., 2014). For each participant, the average displacement of the whole run was calculated and the volume with the largest displacement was identified. No differences were found when average and maximal framewise displacements were compared across groups (*t_48_* = 0.324, *p* = 0.748 and *t_48_* = 1.795, *p* = 0.079, respectively).

### 3.4. Inferential GM models

No GM differences emerged between HC and PD groups from the VBM analysis.

A significant effect of ‘CR’ was found in GM maps (See Supplementary Table S3 in Supplementary material). High-CR individuals had greater volumes in bilateral frontal regions.

No significant main effect of ‘group’ nor a ‘group-by-CR’ interaction emerged on GM maps.

## 4. Discussion

This study tested the hypothesis that in PD patients FC alterations can be detected in large-scale and seed-based resting-state brain networks, and that CR might contribute to modulating these patterns of alterations.

One of the most intriguing results of the present study is that CR may exert a modulatory effect on FC involving basal ganglia and executive-attentional fronto-parietal networks.

We found evidence that CR modulates FC in PD patients by using both an ICA and a seed-based approach. The ICA approach revealed that low-CR PD patients showed lower FC within the basal ganglia (putamen and caudate nucleus) and anterior cingulate. Moreover, the seed-based approach showed lower FC in low-CR PD patients between bilateral frontal and parietal regions, but at the same time stronger FC between the left middle frontal gyrus and medial and superior frontal gyri. The modulation of FC offered by CR supports the hypothesis that lifelong cognitive enrichment may exert a neuroprotective role (Stern, 2002) and may mitigate functional down regulation induced by neurodegeneration (Brayne et al., 2010). An alternative (and largely complementary) explanation would suggest that low-CR individuals may have less resources to cope with the functional changes that occur in the presence of neurodegeneration. It is also worth noting that patients with higher CR showed slightly less motor impairment as evaluated with the MDS-UPDRS-III, suggesting a protective role of CR not only on cognitive but also on motor function, in agreement with previous studies that have reported less severe motor symptoms in PD individuals with higher CR (Guzzetti et al., 2019).

Taken together, the finding of reduced FC in parietal regions and increased FC in prefrontal cortex detected in the low-CR PD patients supports earlier observations from functional neuroimaging studies that report age-related reductions in the activation pattern of posterior regions as well as increases in anterior regions, a potential mechanism at the basis of the “posterior–anterior shift in aging” neurocompensation model (Davis et al., 2008). This model postulates that the recruitment of anterior regions, i.e., prefrontal cortex, might sustain maintenance of cognitive performance in the presence of a reduction in posterior activity (Davis et al., 2008; Park and Reuter-Lorenz, 2009; Grady, 2012). Nevertheless, increased activity in prefrontal regions may also reflect less specific or less efficient functioning, rather than compensation (West, 1996; Morcom and Henson, 2018). Our data are in line with both hypotheses. Further investigations of prefrontal activity and how this correlates with cognitive performance will help shed light on the mechanisms involved.

A main effect of CR was also observed, with low-CR individuals showing more FC between left middle frontal regions and the right cerebellum. Functional neuroimaging studies found that cerebellar regions co-activate with fronto-parietal cortices during cognitively demanding tasks (Balsters et al., 2014) and that cerebellar regions receive input from prefrontal and parietal regions through cortico-subcortical pathways (Krienen and Buckner, 2009; Buckner et al., 2011). This heightened connectivity might represent a compensatory mechanism that may mitigate less efficient neural processing in low-CR individuals. It has been proposed that the cerebellum is intrinsically capable of self-compensation and restoration, and these abilities are referred to as cerebellar reserve (Bordignon et al., 2021).

A final remark concerns structural data. Although no GM differences emerged between HC and PD groups in the VBM analysis, high-CR individuals showed greater volumes in a bilateral frontal cluster. Our findings are consistent with previous studies that have explored the link between CR and structural integrity of older adults’ brains (Anatürk et al., 2018) reporting a positive association between engagement in social-intellectual activities and GM volumes of frontal and temporal areas (Bartrés-Faz et al., 2009; Arenaza-Urquijo et al., 2017). Greater GM volumes in individuals with higher CR may correspond to better tolerance of age-related damage (Stern, 2002; Mortimer et al., 2003; Bartrés-Faz and Arenaza-Urquijo, 2011; Stern et al., 2018), with GM loss concentrated in the prefrontal cortices and subcortical structures, including the hippocampus (Raz et al., 2004; Allen et al., 2005; Driscoll et al., 2009; Taki et al., 2013; Schippling et al., 2017).

Several limitations should be taken into account when interpreting the results of the present study. First, most PD patients were on dopaminergic medication. Studies of drug-naïve patients would exclude the effects of dopaminergic medications on functional examination. Second, we did not test the relationships between FC and clinical profiles inclusive of neuropsychological performance and disease severity, as a very modest difference in MoCA scores was found between the two groups. This modest difference is most likely reflective of the limited psychometric properties of this screening test, since there were no significant differences in scores on the extended neuropsychological assessment between patient subgroups with high and low CR. Although this additional evidence suggests that cognitive variability between our PD subgroups is of marginal relevance to the mechanisms under examination (as cognitive variability also depends on brain networks), this is an aspect that certainly deserves more attention from researchers. Third, pathophysiological factors other than dopaminergic dysfunction might contribute to FC alterations in individuals with PD, e.g., alterations to cholinergic pathways (Bohnen et al., 2022) or TAU pathology (Pan et al., 2021). The link between distinct pathophysiological mechanisms and specific FC abnormalities, however, still needs to be clarified. On this note, we did not include a fine-grained characterization of the profiles of motor symptoms shown by patients (e.g., their type and lateralization), nor did we focus on the impact of reserve on specific cognitive domains such as, for instance, attentional processes and their respective functional (i.e., non data-driven) networks. Future studies will have the opportunity to explore this aspect in more detail, in order to describe the effects of variables of neurological importance (such as CR) as a function of a pathology-informed pattern of FC alterations. Fourth, we used a proxy of CR that did not include other relevant aspects of reserve such as lifelong enriching activities and experiences of leisure time. This information was available as part of the CRIq scale (and all CRIq subscores were significantly correlated with our CR predictor), but missing data prevented us from applying this instrument to the entire cohort. Fifth, although a range of denoising methodologies was applied, it is still possible that the findings might have been, in part, influenced by non-neural sources of signal variability. This methodological consideration is of central importance when PD is studied, as individuals with this condition may show significantly higher levels of in-scanner motion. The framewise displacement values calculated across each individual run, however, were not different between the two diagnostic groups. This indicates that in-scanner motion was not a major cause for concern. It is fair to acknowledge, however, that other, more sophisticated methods (i.e., such as *scrubbing* or *CompCor*) are available to researchers to control for motion and physiological artifacts in a more fine-grained manner. It is also important to point out that, while in-scanner motion has a detrimental impact on signal quality, this effect appears to be more pronounced with magnetic fields of higher strengths (Duyn, 2012). In this respect, although a 1.5 T magnetic field strength provides a lower spatial resolution, it offers the advantage of being less negatively influenced by motion, which is a central issue when PD is studied. Finally, as no body of studies exists on functional brain networks and CR in PD, we decided to rely on a conservative method to define the threshold of statistical significance (i.e., relying on a Family-Wise Error correction). Future studies will have the opportunity to adopt alternative approaches (e.g., threshold-free cluster enhancement methods) to limit false negatives.

The findings of this study complement published literature that has described alterations of resting-state brain activity in PD. The ICA approach revealed that PD patients had significantly less FC within the aDMN in the right primary motor, somatosensory and superior parietal cortices, and within the SMN in the right premotor and supplementary motor areas, regions involved in motor preparation and execution (Lee et al., 1999). Reduced FC at rest in the supplementary motor areas has been previously reported in PD using ICA (Canu et al., 2015; Laganà et al., 2020) and network models based on graph theory (Wu et al., 2009). Conversely, PD patients showed hyper-connectivity in the right inferior frontal gyrus (BA47), anterior cingulate (BA32), and caudate nucleus within the rFPN. Thus, our results on large-scale networks demonstrate that alterations in FC were specifically located in regions considered important hubs of the DMN (Raichle et al., 2001) and FPNs (Japee et al., 2015), areas involved in higher cognitive processes (Andrews-Hanna et al., 2007). A recent meta-analysis (Wolters et al., 2019) reported reduced FC in the DMN and FPNs when PD patients with cognitive impairment were compared with HC. DMN disruption was associated with deficits of perception and executive functions in these patients (Tahmasian et al., 2017). However, abnormal FC of the DMN, significantly correlated with cognitive parameters, was also documented in a rs-fMRI study that included cognitively unimpaired PD patients only (Tessitore et al., 2012), suggesting that DMN alteration may have a role in the development of cognitive decline in PD.

The seed-based approach demonstrated lower FC in PD between the bilateral middle frontal gyrus and bilateral parietal regions, covering the inferior parietal lobule and precuneus, major hubs of the attentional network (Coull, 2004). At the same time, PD patients showed higher FC between the left middle frontal gyrus and a cluster including the posterior cingulate. Recent meta-analyses (Pan et al., 2017; Tahmasian et al., 2017) and reviews (Tessitore et al., 2019) have reported that one of the most consistent findings in PD is an abnormal intrinsic functional pattern in the inferior parietal lobule, as confirmed by ICA, graph theory and ‘amplitude of low-frequency fluctuations’ analyses. Altered function of the rostral inferior parietal cortex (BA40) in PD was also observed in task-related fMRI and PET studies (Samuel et al., 1997; Sabatini et al., 2000). Additionally, exploration of anatomical connectivity through probabilistic tractography indicates that rostral inferior parietal areas are strongly connected with inferior frontal, motor, premotor, and somatosensory regions involved in higher motor functions, whereas caudal inferior parietal areas are predominantly connected with posterior parietal, primary visual and temporal areas typically related to spatial attention and language processing (Caspers et al., 2008).

In conclusion, we found abnormal FC across fronto-parietal circuits in PD patients, and we obtained evidence that CR exerts a relatively strong modulatory effect on FC in executive-attentional networks, typically impaired in PD. Future studies are required to evaluate longitudinal FC modifications to establish if these measures may help prediction of cognitive decline in PD, and if CR is linked to slower disease progression or rehabilitation-related changes. Different trajectories of decline may characterize individuals with high or low CR. Finally, forthcoming research may help gain an understanding of how FC may be the outcome of several cellular and molecular mechanisms related to CR building, including genetic polymorphisms (such as variants of the BDNF gene), epigenetic changes, neurogenesis and synaptic plasticity.

## Data availability statement

The raw data supporting the conclusions of this article will be made available by the authors, without undue reservation.

## Ethics statement

The studies involving human participants were reviewed and approved by IRCCS Don Carlo Gnocchi Foundation Ethics Committee (Ref No. 3_1/7/2015). The patients/participants provided their written informed consent to participate in this study.

## Author contributions

SDT, AV, and MCS conceived the work. FB and SDT collected data. SDT and MDM performed the analyzes. SDT, AV, and MCS interpreted the results. SDT, AV, and MDM wrote the draft of the manuscript. All authors read and reviewed the final version of the manuscript.

## Funding

This research was supported by the Italian Ministry of Health (Ricerca Corrente).

## Conflict of interest

The authors declare that the research was conducted in the absence of any commercial or financial relationships that could be construed as a potential conflict of interest.

## Publisher’s note

All claims expressed in this article are solely those of the authors and do not necessarily represent those of their affiliated organizations, or those of the publisher, the editors and the reviewers. Any product that may be evaluated in this article, or claim that may be made by its manufacturer, is not guaranteed or endorsed by the publisher.

## Footnotes

^1^http://www.restfmri.net

^2^http://www.cs.ucl.ac.uk/staff/g.ridgway/vbm/get_totals.m

^3^http://www.mialab.mrn.org/software/gift

^4^www.thomaskoenig.ch/Lester/ibaspm.htm

^5^www.nature.com/articles/sdata201863

^6^http://imaging.mrccbu.cam.ac.uk/downloads/MNI2tal/mni2tal-m
