# Supplementary material for: Resting-state functional connectivity is modulated by cognitive reserve in early Parkinson’s disease
Source: Front Psychol. 2023 Aug 25;14:1207988. doi: 10.3389/fpsyg.2023.1207988 (PMC10485267; doi:10.3389/fpsyg.2023.1207988)
Supplement: Supplementary file 1 [file Data_Sheet_1.docx]

Supplementary Material

Resting-state functional connectivity is modulated by cognitive reserve in early Parkinson’s disease

Sonia Di Tella, Matteo De Marco, Francesca Baglio, Maria Caterina Silveri, Annalena Venneri

*** Correspondence:** Matteo De Marco: matteo.demarco@brunel.ac.uk

#

# Supplementary Tables

**Table S1.** Demographic and neurostructural characteristics in HC with low cognitive reserve, HC with high cognitive reserve, PD with low cognitive reserve, and PD with high cognitive reserve.

|  | **HC** | | **PD** | |
| --- | --- | --- | --- | --- |
|  | **Low** | **High** | **Low** | **High** |
|  | **[*n* = 10]** | **[*n* = 14]** | **[*n* = 15]** | **[*n* = 11]** |
| **Age (years, *mean* [*SD*])** | 66.32 [7.12] | 63.73 [8.59] | 66.42 [8.74] | 63.64 [7.14] |
| **Education (years, *mean* [*SD*])** | 11.30 [1.64] | 18.07 [2.89] | 10.27 [2.25] | 17.82 [2.60] |
| **Gender (Males/Females, *n*)** | 5/5 | 12/2 | 8/7 | 9/2 |
| **MoCA (*mean* [*SD*])** | 27.12 [2.37] | 25.02 [2.99] | 24.30 [3.71] | 23.57 [2.03] |
| **Cognitive reserve composite index (*median* [*IQR*])** | 6.20 [0.63] | 9.43 [1.12] | 5.53 [1.13] | 9.91[0.83] |
| **Grey matter volume (ml, *mean* [*SD*])** | 609.47 [41.42] | 653.91 [72.70] | 594.04 [62.26] | 644.60 [67.92] |
| **White matter volume (ml, *mean* [*SD*])** | 458.77 [53.22] | 473.12 [65.29] | 457.60 [80.73] | 490.25 [51.34] |
| **Cerebrospinal fluid (ml, *mean* [*SD*])** | 397.85 [97.27] | 441.10 [95.20] | 423.80 [73.72] | 456.18 [84.44] |
| **Total intracranial volume (ml, *mean* [*SD*])** | 1466.09 [116.19] | 1568.13 [133.13] | 1475.44 [156.24] | 1591.04 [135.39] |

IQR: interquartile range; SD: standard deviation

**Table S2.** Clinical and neurocognitive characteristics of the two groups (low and high cognitive reserve) of PD individuals. Adjusted scores are reported (* indicates raw scores, when adjusted scores are not available). (a) Mann-Whitney’s *U* test and (b) Independent samples Student’s *t*-test were used to evaluate group differences. Group averages were all above (or below, for response times and error scores) the cut-offs published by Italian normative studies.

|  | **PD** | | **Group comparison** |
| --- | --- | --- | --- |
|  | **Low** | **High** |  |
|  | **[N = 15]** | **[N = 11]** | **[*p-value*]** |
| **H & Y (median [IQR])** | 1.50 [1.00] | 1.50 [0.50] | 0.245^a^ |
| **MDS-UPDRS III (median [IQR])** | 22.00 [17.00] | 14.00 [19.00] | 0.194^a^ |
| **LEDD (mean [SD])** | 249.07 [208.96] | 246.27 [164.75] | 0.971^b^ |
| **Disease duration (years, mean [SD])** | 3.00 [2.39] | 3.27 [1.79] | 0.753^b^ |
| **Phonological fluency (mean [SD])** | 34.67 [10.37] | 37.40 [7.53] | 0.467^b^ |
| **Semantic fluency (mean [SD])** | 42.27 [9.11] | 43.55 [8.17] | 0.715^b^ |
| **TMT part A (mean [SD])** | 50.00 [25.03] | 40.73 [22.93] | 0.344^b^ |
| **TMT part B (mean [SD])** | 108.60 [112.82] | 86.36 [41.51] | 0.541^b^ |
| **TMT part B-A (mean [SD])** | 63.73 [93.49] | 44.00 [27.76] | 0.506^b^ |
| **Rey-Osterrieth figure copy (0-36) (mean [SD])** | 28.73 [6.42] | 32.91 [3.54] | 0.067^b^ |
| **Rey-Osterrieth figure recall (0-36) (mean [SD])** | 15.10 [5.42] | 15.73 [7.33] | 0.818^b^ |
| **FCSRT IFR (0-36) (mean [SD])** | 29.02 [3.27] | 29.04 [4.61] | 0.994^b^ |
| **FCSRT ITR* (0-36) (mean [SD])** | 35.85 [0.38] | 35.55 [0.93] | 0.335^b^ |
| **FCSRT DFR (0-12) (mean [SD])** | 10.73 [1.14] | 10.11 [1.43] | 0.246^b^ |
| **FCSRT DTR* (0-12) (mean [SD])** | 12 [0.00] | 11.91 [0.30] | 0.341^b^ |
| **FCSRT CSI (0-1) (mean [SD])** | 0.99 [0.03] | 0.81[0.40] | 0.174^b^ |
| **FCSRT number of intrusions (mean [SD])** | 0.08[0.28] | 0.00 [0.00] | 0.369^b^ |
| **Raven coloured matrices (0-36) (mean [SD])** | 29.03 [6.28] | 30.33 [1.76] | 0.488^b^ |

CSI: cueing sensitivity index; DFR: delayed free recall; DTR: delayed total recall; FCSRT: Free and Cued Selective Reminding Test; H & Y: modified Hoehn and Yahr Scale; IFR: immediate free recall; IQR: interquartile range; ITR: immediate total recall; LEDD: Levodopa Equivalent Daily Dose; MoCA: Montreal Cognitive Assessment; SD: standard deviation; TMT: Trail Making Test; UPDRS III: modified version of the Unified Parkinson's Disease Rating Scale – motor part III

**Table S3.** ANCOVA ‘group’ (HC, PD) and ‘CR’ (high, low) results on grey-matter maps. Only the main effect of ‘CR’ is reported. The main effect of ‘group’ and the interaction effect were not significant. *P*-values (*FWE* corrected) lower than 0.05 were considered significant; x, y, z coordinates are in the Montreal Neurological Institute (MNI) space.

|  | **Cluster** | **Peak** | **Peak** | **[mm]** | | | **Side** | **Brain Region** | **BA** |
| --- | --- | --- | --- | --- | --- | --- | --- | --- | --- |
|  | **Extent** | ***T*** | **equiv*Z*** | **x** | **y** | **z** |  |  |  |
| **Main effect of CR: High CR > Low CR** | | | | | | | | | |
|  | 2316 | 4.74 | 4.24 | -16 | 42 | -14 | L | Middle Frontal Gyrus | 11 |
|  |  | 3.88 | 3.58 | -15 | 45 | 12 | L | Medial Frontal Gyrus | 10 |
|  |  | 3.75 | 3.47 | -24 | 23 | -11 | L | Inferior Frontal Gyrus | 47 |
|  |  | 3.74 | 3.47 | -12 | 38 | 0 | L | Anterior Cingulate | 32 |
|  |  | 3.49 | 3.26 | -9 | 39 | 9 | L | Anterior Cingulate | 32 |
|  |  | 3.47 | 3.24 | -10 | 38 | 28 | L | Medial Frontal Gyrus | 9 |
|  |  | 3.39 | 3.18 | -8 | 27 | -6 | L | Anterior Cingulate | 24 |
|  |  | 3.33 | 3.12 | -22 | 25 | -15 | L | Middle Frontal Gyrus | 11 |
|  |  | 3.24 | 3.05 | -30 | 43 | -5 | L | Middle Frontal Gyrus | 11 |
|  |  | 3.23 | 3.04 | 4 | 29 | 4 | R | Anterior Cingulate | 24 |
|  |  | 2.92 | 2.78 | -14 | 48 | 22 | L | Superior Frontal Gyrus | 9 |
|  |  | 2.53 | 2.43 | -36 | 36 | -9 | L | Middle Frontal Gyrus | 11 |

BA – Brodmann area; L: left; R: right
